# Supplementary material for: Non-Immune-Mediated, p27-Associated, Growth Inhibition of Glioblastoma by Class-II-Transactivator (CIITA)
Source: Cells. 2024 Nov 14;13(22):1883. doi: 10.3390/cells13221883 (PMC11593141; doi:10.3390/cells13221883)
Supplement: Supplementary file 1 [file cells-13-01883-s001.zip › Supplementary Figures Cells.pdf]

Supplementary Figure S1

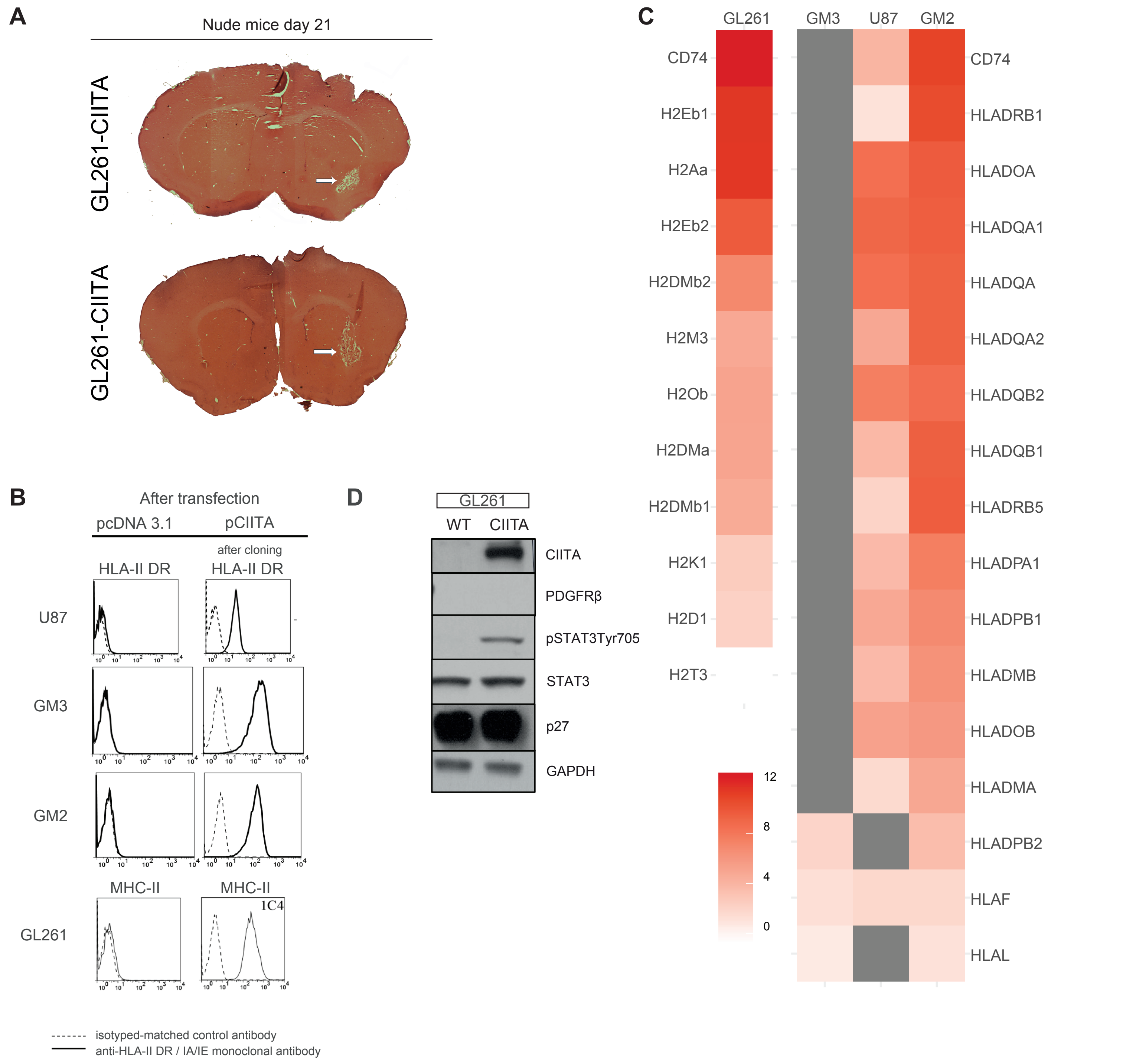

Supplementary Figure S1. A. CIITA-expressing GL261 cells implanted in the brains of athymic nude mice and allowed to grow for 21 days develop only microscopic tumors (Arrows).**B.** FACS data showing MHC-II expression of the four glioma cell lines (human) U87, GM3 and GM2 and (murine) Gl261 after transfection with pcDNA 3.1 (left column) and with the pcDNA3.1-CIITA plasmid (right column). Dashed line representing the isotype-matched control antibody and the continuous line representing anti-MHC-II antibody. **C.** RNA sequencing data showing high expression of genes in the MHC family. **D.** Western blot expression of proteins of interest in GL261, that were also measured in the three human cell lines U87, GM2 and GM3 (Figure 3d). GAPDH here shown as loading

Supplementary Figure S2

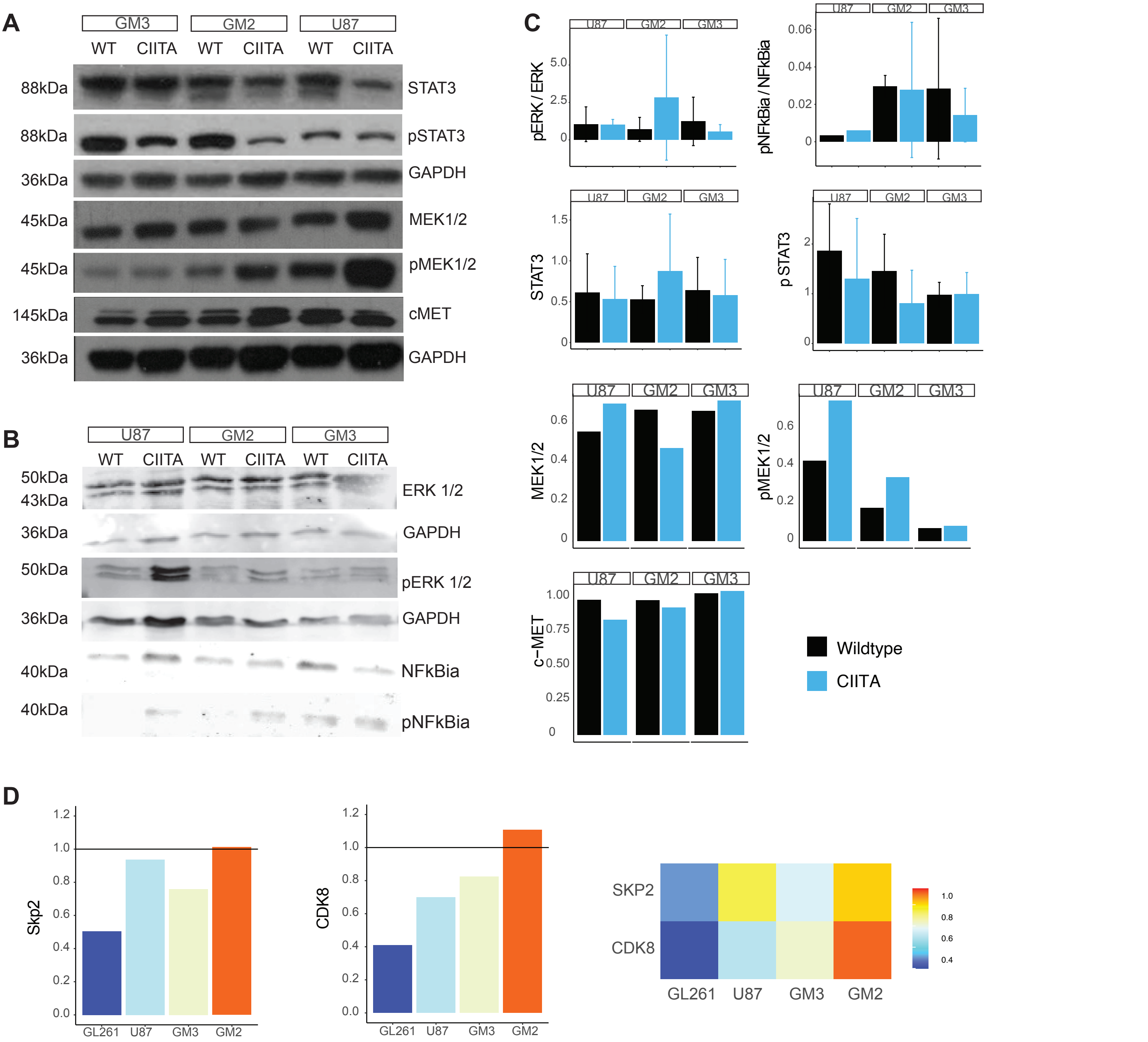

Supplementary Figure S2. A. Western blot showing STAT3, pSTAT3, MEK1/2 and pMEK1/2 expression and **B.** ERK and phospho-rylated (p)ERK expression. **C.** Showing bargraphs representing the analyzed Western blot values, corrected for GAPDH expression as housekeeping gene or for NF-kappa-Bia the phosphorylated fraction divided by the unphosphorylated fraction. **D.** RT2 profiler expression of SKP2 and CDK8, showing upregulation in expression for GM2 anddownregulation in the other cell lines. Below one is downregulation (indicated by a black line), above one represents upregulation. Next to the bargraphs a heatmap of these genes.
